# Uridine as a protector against hypoxia-induced lung injury

**DOI:** 10.1038/s41598-019-45979-2

**Published:** 2019-07-01

**Authors:** Ekaterina V. Rozova, Irina N. Mankovskaya, Natalia V. Belosludtseva, Natalya V. Khmil, Galina D. Mironova

**Affiliations:** 1 Bogomoletz Institute of Physiology, National Academy of Sciences of Ukraine, Bogomoletz street 4, 01024 Kiev, Ukraine; 2 Institute of Theoretical and Experimental Biophysics, Russian Academy of Sciences, Institutskaya street 3, 142290 Pushchino, Moscow region Russia

**Keywords:** Potassium channels, Target identification

## Abstract

The effect of the activation of the mitochondrial ATP-dependent potassium channel (mitoK_ATP_) on the ultrastructure of rat lung in acute hypoxic hypoxia (7% of oxygen in nitrogen, exposure 30 min) was studied. It was shown that uridine, a precursor of the mitoK_ATP_ activator UDP, exerted a protective effect against hypoxic damage to the lung. The administration of uridine to animals prior to hypoxia decreased the number of mitochondria with altered ultrastructure and prevented the hypoxia-induced mitochondrial swelling. Uridine also protected the epithelial, interstitial and endothelial layers of the air–blood barrier from the hypoxia-induced hyperhydration. The protective action of uridine against hypoxia-induced lung injury was eliminated by the selective blocker of mitoK_ATP_ 5-hydroxydecanoate. These data suggest that one of the mechanisms of the positive effect of uridine is related to the activation of the mitoK_ATP_ channel, which, according to the literature and our data, is involved in the protection of tissues from hypoxia and leads to adaptation to it. A possible role of uridine in the maintenance of the mitochondrial structure upon hypoxia-induced lung injury and the optimization of oxygen supply of the organism is discussed.

## Introduction

The mitochondrial ATP-dependent potassium channel (mitoK_ATP_) is known to be present in various tissues of vertebrates, as well as in invertebrates, fungi, and plants^1–3^. Recently, much attention has been paid to the crucial role of the channel in the protection of tissues against hypoxia^4,5^.

It has been established that the development of hypoxia in the organism caused by various factors, including the extremely reduced O_2_ concentration in the inspired air, leads to the disorders of ultrastructure of tissues and, consequently, the disturbance of their functioning. In this case, there is a pronounced hyperhydration of tissues and, especially, the biological barriers due to changes in the permeability of the cytoplasmic membrane^5–7^. One of the first targets in the cell under hypoxic conditions is mitochondria, which largely lose their ability to produce macroergs^8,9^.

Earlier it was shown that mitoK_ATP_ involved in the maintenance of potassium ion homeostasis played a significant role in the functioning of mitochondria, and the its activation protected tissue against hypoxic damage^10^. The introduction of pharmacological mitoK_ATP_ activators before the onset of hypoxia or ischemia had an effect similar to preconditioning, and the inhibitors of the channel abolished the positive effects of preconditioning^11,12^.

We found that uridine-5′-diphosphate (UDP) is the effective metabolic activator of mitoK_ATP_^13^ and its precursor uridine has the anti-ischemic and antiarrhythmic effects^14^. It was shown that the uridine maintained the energy balance and antioxidant status of the myocardium under conditions of acute coronary insufficiency^15^. However, it remains unknown whether similar effects manifest themselves in other organs, in particular the lung, which provides an oxygen uptake requiring high-energy expenditure, especially on exposure to low environmental pO_2_.

The aim of this work was to study the influence of uridine on the ultrastructure of the lung tissue in acute hypoxic hypoxia and to identify the mechanism of its possible protective effect against hypoxic lung injury. The results obtained showed that uridine has a potent protective effect against hypoxic damage not only in the myocardial tissue, as it was found previously^10^, but also in the lung tissue. The mechanism of action of uridine can be associated with the activation of ATP-dependent potassium transport in mitochondria.

## Results

### Changes in the ultrastructure of the air–blood barrier in acute hypoxic hypoxia and their correction by uridine

**Figure 1:**
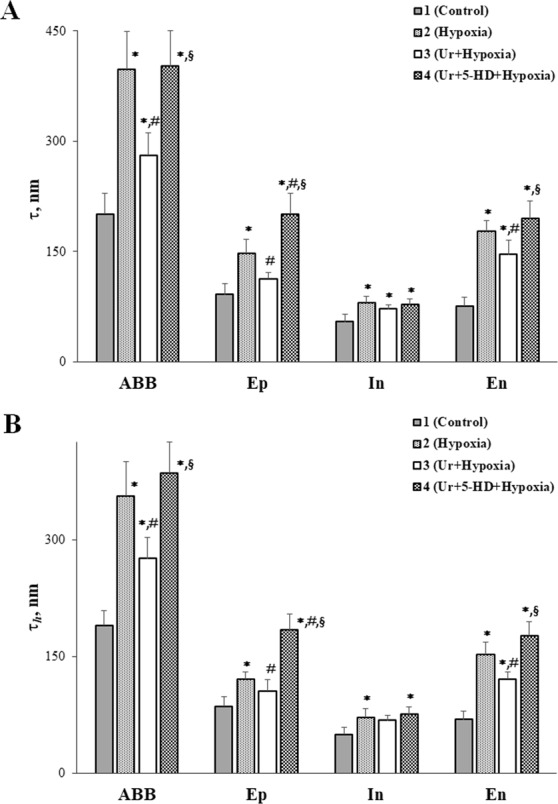
Effect of mitoK_ATP_ modulators on the average arithmetic (*τ*) (**A**) and harmonic (*τ*_h_) (**B**) thicknesses of the air-blood barrier of the lung and its individual layers in acute hypoxic hypoxia. Abbreviations: ABB, the air-blood barrier; Ep, the epithelial layer of the ABB; In, the interstitial layer of the ABB; En, the endothelial layer of the ABB; Ur, uridine; 5-HD, 5-hydroxydecanoate. Four experimental groups were included: 1 – non-treated rats (Control); 2 – rats exposed to 30 min acute hypoxic hypoxia (7% O_2_) (Hypoxia); 3 – rats treated with uridine (0.3 mg/100 g) 30 min prior to hypoxic exposure (Ur + hypoxia); 4 – rats treated with the selective inhibitor of mitoK_ATP_ 5-hydroxydecanoate (5-HD, 0.05 mg/100 g) 10 min after the administration of uridine and 20 min prior to hypoxic exposure (Ur + 5-HD + hypoxia). There were six rats in each group, and 80 replicates per rat. Values are means ± SD (*n* = 6, *a* = 80, where *n* - the number of experiments; *a* – the number of calculated fields). *Statistically different from the control values (*p* < 0.05). ^♯^Statistically different from the values of the 2nd group (*p* < 0.05). ^§^Statistically different from the values of the 3d group (*p* < 0.05).

**Figure 2:**
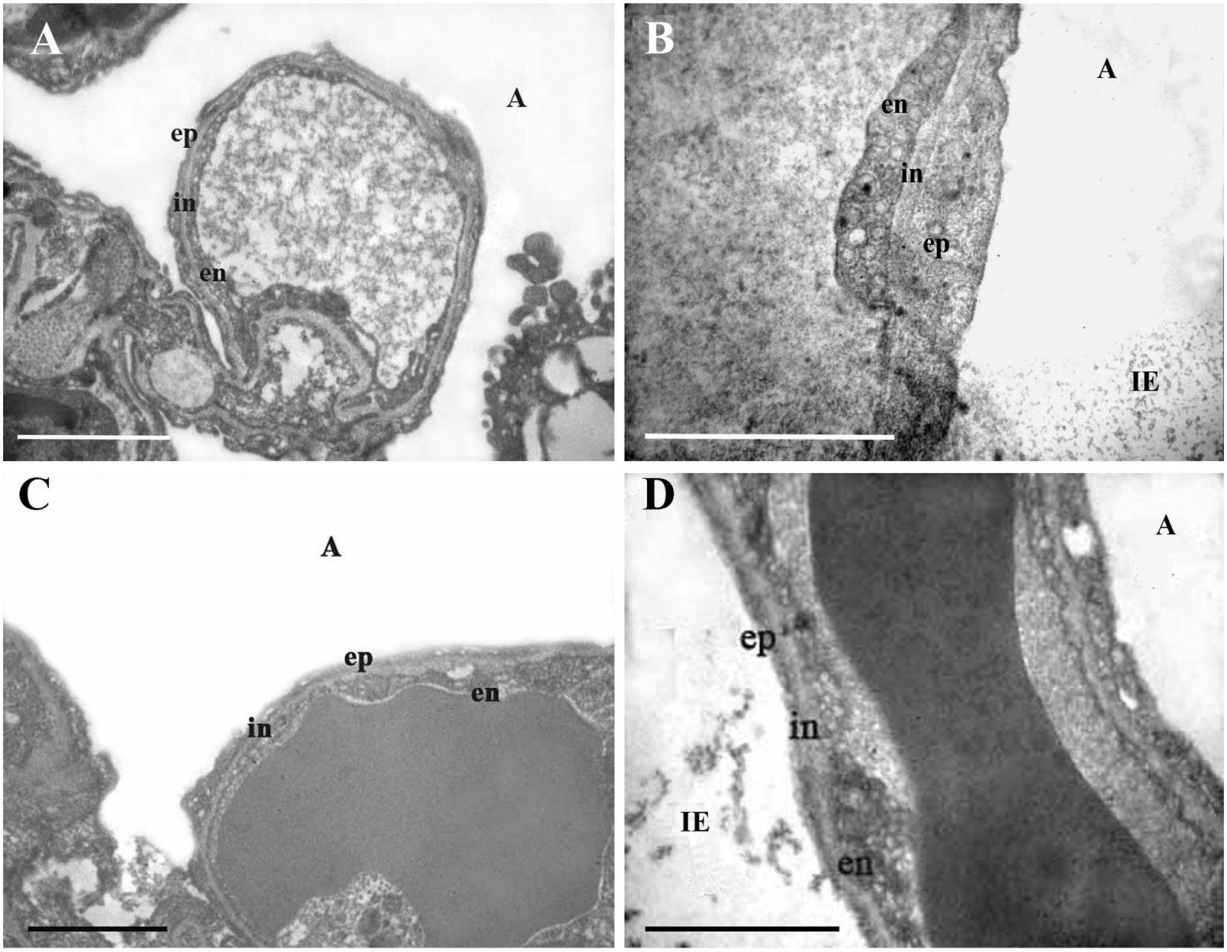
Ultrastructure of the ABB of the lung of rats in four experimental groups: control (**A**), hypoxia (**B**), uridine + hypoxia (**C**), and uridine + 5-HD + hypoxia (**D**). Abbreviations: A, the alveolus; ep, the epithelial layer of the ABB; in, the interstitial layer of the ABB; en, the endothelial layer of the ABB; IE, intraalveolar edema. The groups used in this experiment were the same as those in Fig. 1. There were six rats in each group. All animals were analyzed individually and simultaneously. Results are representative of six independent experiments. Scale bar 1 μm.

Figure 1 shows that acute hypoxic hypoxia (hereafter referred to as “hypoxia”) resulted in a sharp increase in the average arithmetic (*τ*) and harmonic (*τ*_h_) thicknesses of the the air–blood barrier of the lung (ABB). The increase in *τ* was more pronounced than in *τ*_h_. The thickening of the ABB occurred in all its individual layers. The average arithmetic thickness of the epithelial layer increased by 61%; of the interstitial layer, by 47%; of the endothelial layer, 2.3 times. The average harmonic thickness of the epithelial layer increased by 41%; of the interstitial layer, by 44%; and of the endothelial layer, 2.2 times. Thus, hypoxia resulted in the intraalveolar edema of all the layers, which is well defined in electron microscopy images (Fig. 2A,B).

In the presence of uridine, the thickness of the epithelial layers of the ABB became equal to that in the control group (Figs 1A,B and 2C). The *τ* and *τ*_*h*_ values of the endothelial layer of the ABB after the preliminary administration of uridine to animals was reduced by 19% compared to those in hypoxia (Fig. 1). At the same time, uridine *in vivo* did not significantly change the thickness of the interstitial layer of the ABB.

### Changes in the ultrastructure of rat lung mitochondria in acute hypoxic hypoxia and their correction by uridine

**Figure 3:**
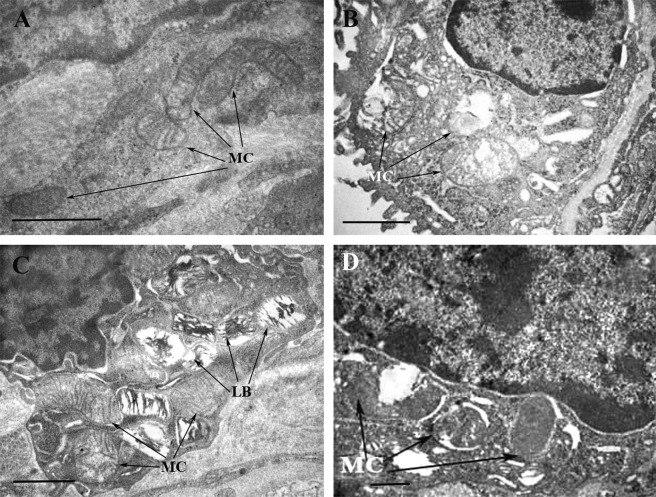
Ultrastructure of the rat lung mitochondria in four experimental groups: control (**A**), hypoxia (**B**), uridine + hypoxia (**C**), and uridine + 5-HD + hypoxia (**D**). Abbreviations: MC, mitochondria; LB, lamellate bodies. The groups used in this experiment were the same as those in Fig. 1. Results are representative of six independent experiments. Scale bar 0.5 μm.

We also examined the effect of exposure to acute hypoxic hypoxia on ultrastructure of mitochondria in lung cells (Fig. 3). The following structural features of rat lung mitochondria were found: the swelling of the mitochondrial matrix of different degree, partial or complete vacuolization, the disorders in crista arrangement, destruction of the mitochondrial membranes, mainly of the inner, and sometimes of the outer ones.

**Figure 4:**
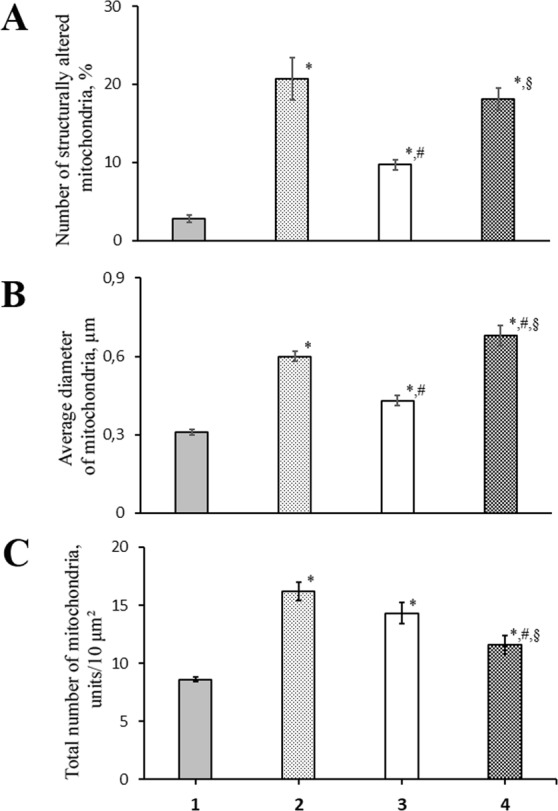
Morphometric analysis of rat lung mitochondria in acute hypoxic hypoxia in the presence and absence of mitoK_ATP_ modulators: number of structurally altered mitochondria (**A**), average diameter of mitochondria (**B**); total number of mitochondria. (**C**) Four experimental groups were included: 1 – non-treated rats (Control); 2 – rats exposed to 30 min acute hypoxic hypoxia (7% O_2_) (Hypoxia); 3 – rats treated with uridine (0.3 mg/100 g) 30 min prior to hypoxic exposure (Ur + hypoxia); 4 – rats treated with the selective inhibitor of mitoK_ATP_ 5-hydroxydecanoate (5-HD, 0.05 mg/100 g) 10 min after the administration of uridine and 20 min prior to hypoxic exposure (Ur + 5-HD + hypoxia). There were six rats in each group, and 80 replicates per rat. Values are means ± SD. ^*^Statistically different from the control values (*p* < 0.05); ^♯^Statistically different from the values of the 2nd group (*p* < 0.05); §Statistically different from the values of the 3d group (*p* < 0.05).

It should be noted that, in our experiments, the exposure to hypoxia initiated an adaptive response at the cellular level, in particular, mitochondrial morphogenesis, so that the total number of lung mitochondria increased by 88.4% (Fig. 4). However, there was also a sevenfold increase in the number of structurally damaged organelles.

### Elimination of the protective effect of uridine by the selective inhibitor of mitoK_ATP_ channel 5-hydroxydecanoate

The protective effect of uridine on dramatic ultrastructural changes in mitochondria and the ABB revealed in hypoxia can be associated with the activation of mitoK_ATP_. As one can see from Figs 2D and 3D, the administration of the selective inhibitor of mitoK_ATP_ 5-hydroxydecanoate (5-HD) (0.05 mg/100 g) prior to the injection of uridine abolished almost all of the observed effects of uridine. First, this concerns the development of the ABB edema (Fig. 1). The electron microscopy study and further morphometric measurements showed that, in the presence of 5-HD, the thickness of the ABB of the lung and all its individual layers was significantly greater than in the control (Figs 1 and 2D) and was close to the values observed in hypoxia. It should be emphasized that the hyperhydration of the epithelial layer in this group of experiments was even more pronounced than under hypoxia: *τ* and *τ*_h_ increased by 36% and 52% correspondingly as compared with the values of the 2nd group (Fig. 1).

At the same time, structural changes in the lung mitochondria of rats treated with 5-HD 10 min after the administration of uridine and 20 min prior to hypoxic exposure were similar to those occurring during the ordinary response to hypoxic exposure: the amount of structurally damaged mitochondria and their mean diameter did not significantly differ from those observed in hypoxia, and the mitochondrial morphogenesis was even less pronounced (Fig. 4). It should be noted that 5-HD alone did not significantly change the control values of the thickness of the ABB of the lung, as well as mitochondrial ultrastructure (data not shown).

## Discussion

In this study, we found that hypoxia leads to a significant rise in the thicknesses of all individual layers of the ABB (Fig. 1), which may be associated with the hyperhydration of the ABB due to an increase in the permeability of cytoplasmic membranes^7,16^. The increase in *τ* was more pronounced than in *τ*_h_, which indicates a relatively uniform distribution of thickened and thin sections of the ABB in hypoxia^17^. The ultrastructural disturbances of the ABB led to the intraalveolar edema, which is easily detectable by electron microscopy (Fig. 2A,B).

The morphometric analysis also revealed a hypoxia-induced increase in the total number of rat lung mitochondria (Fig. 4C). According to our earlier data, a rise in the number of mitochondria may be related to the adaptation in tissues in response to hypoxic exposure^18^. At the same time, the total number of structurally damaged mitochondria and their average diameter were found to rise, which is a consequence of common pathological changes in the permeability of both cytoplasmic and intracellular membranes (Figs 3A,B and 4A,B). The observed disorders of the ABB ultrastructure are typical for hypoxia^19^. It was assumed that the pronounced swelling of mitochondria indicated their irreversible damage, which further could lead to cell death through the necrotic pathway^20,21^.

Since the aim of our research was to find a natural modulator of hypoxia, and taking our earlier data on the protective effect of uridine during myocardial ischemia into account^14,15^, we decided to use this modulator to protect lung tissue from hypoxia-induced damage.

The preliminary administration of uridine to animals before hypoxia exposure had a protective effect on the lung mitochondria ultrastructure, contributing to the prevention of the ABB hyperhydration (Fig. 1). The endothelial layer of the ABB in the uridine-treated animals was hydrated to a significantly lesser degree than in hypoxia without uridine, which was clearly seen on electron microscopy images (Fig. 2C).

The injection of uridine decreased the number of mitochondria with a disturbed structure by 53% compared to that in hypoxia (Figs 3C and 4A). Furthermore, uridine prevented excessive swelling of the organelles: their diameter exceeded the control values by 39%, while in hypoxia it increased by 90% compared to the control (Fig. 4B). However, uridine did not significantly reduce the total number of mitochondria, which increased in hypoxia (Fig. 4C). This confirms our earlier suggestion that an increase in the number of mitochondria is an adaptive response of the organism to oxygen deficiency^18^.

We assume that the less pronounced changes in mitochondrial ultrastructure under hypoxia in the presence of uridine are related to an activation of the mitoK_ATP_ whose important role in the protection of heart tissue against ischemia is widely discussed in the literature^10,11,14^. It is known that the administration of uridine increases the concentration of UDP in tissues. The intraperitoneal injection of uridine (30 mg/kg) to rats was found to increase UDP approximately two times after 30–60 min in spleen and heart tissues^22^, which is sufficient for the channel activation, since the effective UDP concentration is 20 μM^13,23^. This suggestion was confirmed by the experiments with 5-HD, a selective inhibitor of mitoK_ATP_^24,25^. The administration of 5-HD prior to the injection of uridine abolished almost all of the effects of uridine. It eliminated the positive effect of uridine on both the morphofunctional state of the ABB (Figs 1 and 2D) and on the morphometric characteristics of mitochondria in the lung tissue (Figs 3D and 4). The fact that the inhibitor slightly enhanced the ultrastructural damage of mitochondria in comparison with that in hypoxia (group 2) may indicate initial activation of mitoK_ATP_ during a half-hour hypoxia. A similar effect of 5-HD was also observed in a model of physiological hypoxia during long-term swimming of rats with low resistance to oxygen deficiency^26^. Under these conditions, adaptive (constructive) processes in heart tissue can mainly occur^18^.

Thus, the results suggest that one of the mechanisms by which uridine protects against hypoxia-induced lung injury is its activation effect on the mitoK_ATP_. According to the literature and our data, the channel can be involved in the protection of tissues from hypoxia and in adaptation to it by decreasing the level of H_2_O_2_ in mitochondria, which prevents the development of oxidative stress^10,11,15,27^. Thus, this molecular mechanism can be involved in the elimination of hypoxia-induced damage to cellular structures, including, in particular, the ABB.

The results suggest that uridine has a potent protective effect against hypoxia-induced damage to lung tissue. The antihypoxic properties of uridine may play an important role in the optimization of oxygen supply of the organism. The results of the study show promise for the application of uridine in the treatment of diseases associated with the development of hypoxia.

## Methods

### Object of the study

The study was carried out on four experimental groups of animals (adult male Wistar rats weighing 220–250 g): 1st group (control) – non-treated rats; 2nd group (hypoxia) – rats exposed to 30 min acute hypoxic hypoxia (7% O_2_); 3rd group (uridine + hypoxia) – rats injected i/v 30 min prior to hypoxic exposure with uridine (AppliChem, Germany) from which the mitoK_ATP_ activator uridine-5′-diphosphate (UDP) was formed in the tissue at a dose of 0.3 mg/100 g of body weight as described earlier^14^; 4th group (uridine + 5-HD + hypoxia) – rats injected i/v 10 min after the administration of uridine (i.e., 20 min before hypoxia) with the selective inhibitor of mitoK_ATP_ 5-hydroxydecanoate (5-HD, Schering AG, USA) at a dose of 0.05 mg/100 g of body weight as described earlier^14^. There were six rats in each group.

The study with laboratory animals was carried out in accordance with the European Convention for the Protection of Vertebrates used for experimental and other purposes (Strasbourg, 1986) and the principles of the Helsinki Declaration (2000). All the protocols were approved by the Institute of Theoretical and Experimental Biophysics Ethics Committee (Order No. 173/k of 03.10.2011, Protocol No. 02 of 01.03.2018).

### Modeling of the hypoxic conditions

The animals were placed in a sealed 10-L chamber and exposed to a gas mixture containing 7% of oxygen in nitrogen for 30 min. The gas mixture was continuously fed by a modified Atman-air pump (CPR), while CO_2_ was absorbed by dried soda lime. The choice of the gas mixture of this composition was dictated by the fact that exactly this low oxygen concentration in the inspired air makes it possible to determine the limits of the adaptive capabilities of the organism, both at the systemic and tissue levels^28^.

### Electron microscopic research

The animals were anesthetized with ether and then decapitated. The pieces of tissue were taken from the identical parts of the lower lobes of both lungs using the conventional approach. The samples were fixed by 2.5% glutaraldehyde solution (pH 7.3) and then by the Caulfield reagent (2% osmium tetroxide solution, pH 7.3) (Sigma, USA). The dehydration of the tissue samples was performed using graded alcohols of increasing concentrations, absolute alcohol and acetone; the subsequent embedding of the samples into epon araldite (Fluka, Switzerland) was carried out according to a generally accepted procedure^29^. Ultrathin sections of 40–60 nm thick were contrasted with a 1% uranyl acetate and plumbum citrate solutions (Sigma, USA) by the Reynolds method^30^ and examined under an electron microscope JEM 100CX (Japan).

### Morphometric study

On electron micrographs, the mean arithmetic (*τ*) and harmonic (*τ*_h_) thicknesses of the air–blood barrier of the lung (ABB) and its individual layers were evaluated by the method of Chalkley *et al*. in the modification of Weibel^17,31^. In accordance with the methodology, a template containing 15 identical lines of a particular length (3, 5, or 7 mm), evenly distributed in 3 directions, and 30 end points was used to calculate morphometric changes. Using the principle of randomness, the template was superimposed on a sample under study. On each photograph, measurements were made at 4 points at 4 positions of the template, i.e. 4 values of *τ* and *τ*_*h*_ were obtained.

For each exposure, 20 electron microscope images were analyzed and, consequently, 80 calculations of the air–blood barrier thicknesses (*a*) were made.

The average arithmetic thickness *τ* which characterizes the mass of the tissue between the units of area measurement of the outer and inner surfaces of the biological barriers was calculated by the formula:

$$\tau =(l\times P)/2({n}_{i}+{n}_{e}),$$

where *l* is the distance between the end points of the measuring line; *P* is the number of end points of the measuring lines located on the tissue barrier; *n*_*i*_ is the number of intersections of the measuring lines with the inner surface of the barrier; and *n*_*e*_ is the number of intersections of the measuring lines with the outer surface of the barrier.

The average harmonic barrier thickness *τ*_*h*_ is the total effective thickness of the tissue structure under consideration, with allowance for the diffusion resistance, and is the arithmetic mean of the reciprocal of *τ*. The average harmonic thickness was determined on the same micrographs as the arithmetic mean^17,31^. The morphometric characteristics of mitochondria were determined on 130–150 fields for each exposure using the computer program for morphometric analysis Image Tool Version 3 (USA).

### Statistical data analysis

Statistical analysis of the results was carried out using the Statistica 10.0 software. Initially, we checked the normality of data distribution using the Shapiro-Wilkie criterion^32^. The data completely fit to the normal distribution due to a large array of measurements. Then, the statistical significance of differences between the groups was evaluated using a *Post Hoc* analysis with the Newman-Keuls multiple comparison test. The differences were considered statistically significant at *p* < 0.05. The results are presented as mean ± standard derivation (m ± SD).
